# Trait Associations in Diversity Panels of the Two Common Bean (*Phaseolus vulgaris* L.) Gene Pools Grown under Well-watered and Water-Stress Conditions

**DOI:** 10.3389/fpls.2017.00733

**Published:** 2017-05-09

**Authors:** Asrat Asfaw, Daniel Ambachew, Trushar Shah, Matthew W. Blair

**Affiliations:** ^1^International Institute of Tropical AgricultureAbuja, Nigeria; ^2^South Agricultural Research InstituteHawassa, Ethiopia; ^3^Department of Agricultural and Environmental Sciences, Tennessee State UniversityNashville, TN, USA; ^4^International Institute of Tropical AgricultureNairobi, Kenya

**Keywords:** drought stress, matric correlation, multiple adaptive traits, trait interrelation, genetic divergence

## Abstract

Common beans are a warm-season, food legume cultivated in areas prone to water limitation throughout their growing season. This study assessed the magnitude and pattern of trait associations for a total of 202 common bean genotypes divided into panels of 81 Andean and 121 Mesoamerican gene pool accessions grown under contrasting treatments of well-watered, non-stress, and water-limited, terminal drought-stress conditions. Linear correlation, complex path coefficient, and genetic divergence analyses were used to dissect the relationship dynamics between traits and the relative contribution of adaptive traits to differentiation among gene pools and genotypes based on drought stress. Drought severity level for the trial was high and created the ideal condition to reveal genotypic differences, as seen by the differential response of the genotypes for the various traits measured. The value for phenotypic coefficients of variation for all traits was higher than the corresponding genotypic values. Seed yield had positive and strong genotypic and phenotypic correlation with pods per plant across gene pools and stress levels. The overall amount of genetic correlation was greater than the corresponding phenotypic correlation matrix for all the traits within the gene pool and across stress levels. Moreover, the results depicted the phenotypic correlation as equal or better than its genotypic counterpart in estimating drought tolerance in common bean plants. Clustering analysis with Mahanalobis's coefficient of generalized distance grouped genotypes with a differential level of drought adaptation into different classes within each panel. This indicates drought tolerance involves different mechanisms of plant response and is present separately in each gene pool panel. Pods per plant, seed weight, pod partitioning index, and harvest index are useful selection objectives to improve drought adaptation in common bean, but must be differentially weighted in each gene pool. The analysis of genetic variation and association between adaptive traits on the two panels provided useful insights on which traits could be used to improve common bean adaptation to low water availability during the growth season.

## Introduction

Common bean (*Phaseolus vulgaris* L.) is one of the important grain legumes in food and agricultural systems of Africa and Latin America. It is widely cultivated throughout the tropics for its edible green leaves, green pods, mature or immature seeds for human food and straw as fodder for animal feed (Broughton et al., 2003; De Luque et al., 2014). Despite its important role in agri-food systems of the tropics, common bean production in many regions is being challenged by drought stress that is part of the many calamities of climate change and a frequent abiotic constraint in East Africa (Wortmann et al., 1998; Asfaw and Blair, 2014). Drought stress, which refers to inadequate water availability in quantity and distribution during the life cycle of the crop, is the most important production risk for common bean worldwide (Beebe et al., 2013). Drought stress potentially affects over 60% of the common bean production area globally in any given year, including vast areas in eastern and southern Africa, Latin America and the Caribbean (White and Singh, 1991; Thung and Rao, 1999). Drought is the most significant contributor to yield reduction and seed insecurity in the rain-fed common bean production systems of most developing countries, where there are few investments in irrigation to raise a good common bean crop and where bean growing is characteristic of poverty hot spots (Asfaw et al., 2013).

Drought during a planting season has a multi-faceted effect on common bean growth and performance whenever and wherever it occurs. This abiotic stress causes poor root growth and development, reduced P uptake and N fixation, poor rates of photosynthesis, low biomass production, and inefficient partitioning (Acosta-Gallegos and Kohashi-Shibata, 1989; Singh, 1995; Serraj and Sinclair, 1998; Asfaw and Blair, 2012; Asfaw et al., 2012). Drought also results in flower abortion, pod drop, reduced seed filling, lower grain weight and ultimately in lower seed yield (Beebe et al., 2013; Asfaw and Blair, 2014; Mukeshimana et al., 2014). Drought stress can cause 20–90% common bean yield reduction in farmers' fields which in the worst scenario could be up to 100% yield loss (Ambachew et al., 2015). Fortunately, the drought problem in common bean production can be mitigated.

Development and deployment of climate resilient varieties of common bean that can stabilize or increase seed yield under drought stress while meeting the producer and consumer demands would position this legume to have a greater role in improved livelihoods in the tropics and elsewhere. These crop improvements require a better understanding of the dynamics of the drought tolerance mechanisms functioning during the exposure of common bean to differential water availability and their relative contributions to stabilized or increased seed yield (Ambachew et al., 2015). Improved seed yield under variable stress factors is the result of not only a single trait but rather a cumulative function of many interdependent plant traits (Asfaw et al., 2012). The genetic and phenotypic relationships of traits are of practical interest in breeding programs as selection for one trait may cause improvement or deterioration in associated trait (Baker, 1986) and simultaneous selection of multiple traits would be desirable if possible.

Accurate assessment of the interconnectedness of plant traits under exposure to variable and low water availability and their contribution to seed yield formation is a key factor for making breeding progress in drought stress. Different methods have been extensively employed in many crops including common beans to understand and exploit the genetic basis of relationship between overall and component production traits in the breeding of new varieties. These methods include simple correlation and causation assessment using linear correlations and regression or complex path coefficients and principal component analysis (Wright, 1923; Li, 1956; Sharma, 1995; Falconer and Mackay, 1996; Waitt and Levin, 1998; Yan and Rajcan, 2000; Önder et al., 2013). Despite the potential of these methodologies for dissection of the genetic basis of trait associations and determining the selection strategies in breeding programs, reports in the literature on its application to common bean under drought conditions are scanty. Previous trait association studies on drought affected common bean plants are primarily based on breeding populations or advanced lines and do not provide both genotypic and phentoypic correlation of traits together. Therefore, few studies in common beans report the extent to which phenotypic correlations between traits reflect its genotypic counterpart over stress factors.

While most drought studies in common bean have used limited genetic diversity found in one or two races of the crop, in this study we develop a germplasm panel for the dissection of drought tolerance traits in all types of the grain legume. Our overall goal was to assess the inter-relationships of multiple drought adaptive traits in two major gene pools of common bean using two reference diversity panels for Andean and Mesoamerican beans from two previous studies grown in contrasting water availability: one with high and one with low total rainfall during the growing season in southern Ethiopia. The reference panels were expected to be informative based on the fact that the different gene pools are the result of separate domestication process in common bean (Blair et al., 2009) and therefore should not be expected to have similar genotypic and phenotypic trait correlations. This study compared and contrasted the pattern and structure of trait correlations and differentiation in the two gene pools using correlation, path and genetic variability analyses. Genetic correlation matrices were compared with their phenotypic counterparts to assess how close the correlation estimates were for traits in the two different gene pools and across stress levels. Such analysis could yield significant additional insights on the organization of trait interrelationships in the common bean plants and would inform a breeding program on how to consolidate the main traits together into new varieties for tropical environments like the one used in the study.

## Materials and methods

### The study material and trial design

The study was conducted at Hawassa, in South Nations, Nationalities and Peoples Regional State (SNNPRS), Ethiopia. Hawassa is located at the 7°03′N latitude and 38°30′E longitude at an elevation of 1700 meter above sea level. The soil at this site is a sandy loam (Flavisol, FAO classification) with pH 7.0 which is characterized by low water retention capacity and which is quick to drain under normal rainfall and drainage system. Hawassa has a bimodal rainfall pattern with an extended period of wet season from March to October with mean monthly rainfall varying from 18 to 128 mm and annual maximum and minimum temperatures ranging from 24 to 29°C and 9.5 to 14.1°C, respectively, based on the long-term weather record from Hawassa station. A quick onset of permanent plant wilting in cases of rainfall deficit during the season due to intense evaporation and percolation is the main feature of the experimental site.

The study material consisted of 202 common bean accessions, of which 81 genotypes were from the Andean gene pool and 121 genotypes were from the Mesoamerican gene pool. Both diversity panels represented accessions held at the International Centre for Tropical Agriculture (CIAT) as described in Pérez et al. (2011) and Simbarashe (2013). The genotypes from the two gene pools were evaluated in separate trials planted in a lattice design with three replications. The trials were planted from July-October 2009 season using two different sowing dates: one that was early to avoid drought and one that was late to expose the crop to terminal drought. These differing conditions created two treatments, one with high and one with low total seasonal rainfall during the growing season. The trials were planted in four rows of 3 m long plots using 60 cm between-row and 10 cm within-row spacing. Across all trials, diammonium phosphate fertilizer was side drilled into each row in a plot at the rate of 100 kg ha^−1^ at planting. A seed dressing of fungicide (Benomyl, a systemic benzimidazole) and insecticide (Thiamethoxam, a systemic insecticide in the class of neonicotinoids) was applied to reduce the effect of diseases and insect pests prevalent during both the rainy and dry season planting for valid testing of the drought resistance of the germplasm. Therefore, disease and insect pressure was insignificant during both trials and for the season overall. The experimental plots were hand-weeded before flowering and again when needed. Total rainfall was recorded on a daily basis with a rain catchment system. Drought-stressed and non-stressed conditions were considered separate experiments for analysis.

### Plant trait measurements

Multiple plant traits were measured using non-destructive and destructive sampling at different crop growth stages based on accepted common bean drought trait ontology (http://www.cropontology.org/ontology/CO_335/Common%20bean). Traits were: (1) days to flowering (DF) based on number of days from sowing to when 50% of plants in a plot opened at least one flower; (2) days to physiological maturity (DPM) based on number of days from sowing to when the first pod begins to discolor in 50% of the plants; (3) non-destructive SPAD chlorophyll meter reading (SCMR) measured at mid-pod filling stage, about 1 month after flowering on 10 fully expanded young leaves of five comparable plants in each plot using a portable SPAD-502 chlorophyll meter (Minolta Camera Co., Ltd., Japan). Other measurements were recorded on five comparable plants per plot sampled at mid pod-filling and at harvest using sampling described in Asfaw et al. (2012) or Beebe et al. (2013) including; (4) pod partitioning index (PPI, %) determined as the ratio of dry weight of pods at harvest over dry weight of total biomass at mid-pod fill multiplied by 100; (5) pod harvest index (PHI, %) determined as the ratio of dry weight of seed over dry weight of pod at harvest multiplied by 100; and (6) harvest index (HI, %) determined as the ratio of seed dry weight at harvest over dry weight of total biomass at the mid-pod fill stage multiplied by 100. The biomass sampled from each plot at mid-pod fill and harvest were hand-separated into leaves, stems, pods and seeds and were oven-dried at 80°C for 48 h. The dried samples were weighted for determination of the indices. Meanwhile, we also measured four additional traits, namely trait (7) pods per plant (PDPL); trait (8) seeds per pod (SDPD); trait (9) seed weight (100SW, g/100seed); and trait (10) seed yield per hectare (YLDH, kg/ha) all recorded at harvest. PDPL was recorded by counting the average number of pods on five randomly selected plants in a plot. SDPD was recorded by counting the average number of seeds on 10 randomly selected pods from five plants in a plot. Seed weight was measured as weight in grams of 100 seeds and seed yield per hectare as weight (kg) of seed harvest divided by the effective plot in m^2^ and multiplied by 10. Seed yield was on a plot basis after the grain was sun-dried to maintain the seed moisture at 12 % and recorded using a sensitive digital balance (A&D FX3000i, 3,200 × 0.01 g). Simple sampling was also used to determine 100SW as the weight of 100 seeds randomly sampled from the plot yield. In addition to the individual genotype measurements, drought intensity index (DII%) calculated as DII = [1 − (Y_ds_/Y_ns_)] 100, where Y_ds_ and Y_ns_ are the mean seed yield of all genotypes under drought stress and non-stress treatments, respectively (Fischer and Maurer, 1978), was used to quantify the severity of drought stress on seed yielding potential of the genotypes. Drought intensity index was also calculated for other traits measured in the trials to quantify the drought stress effect on trait expression compared with their non-stress counterpart. We also estimated the drought response index (DRI) for individual genotypes as suggested by Bidinger et al. (1987) by adjusting genotype capacity for seed yield potential and growth duration. For the drought response estimation, we regressed stress seed yield on potential (non-stress) yield and phenology (days to flowering and days to physiological maturity under non-stress) and calculated standardized residual from the regression estimation as the difference between measured seed yield under drought stress and predicted stress seed yield divided by the standard error. The standardized residual value was used to define the genotype as drought resistance (a positive deviation from the regression line) or susceptible (a negative deviation), independently of the effect of potential yield or growth duration on its seed yield under stress. The response of a genotype to drought was assumed zero (no response) if the difference between predicted and measured seed yield does not exceeds the experimental error.

### Statistical analysis

The data recorded for each trait per trial were initially subjected to mixed model analysis using the program Genstat v. 12.1 (Payne et al., 2007) to test the genotype differences in respective trials. We fit the data to a linear mixed model following Gilmour et al. (1997) using Genstat, where replication and genotypes were fixed and block was a random factor. We also used flowering and maturity time as a covariable in the model to correct their effect on seed yield. The restricted maximum likelihood (REML) procedure was used to estimate the variance components and residual variances. The model used was:

(1) $$\begin{array}{r} Y_{ilm}=\;\mu +g_{i\;}+r_{l\;}+\;b_{m\;}+\varepsilon_{ilm\;} \end{array}$$

where *Y*_*ilm*_ was plot mean performance of a certain genotype *i*, in replication *l* and block *m*, μ the overall mean, *g*_*i*_ the effect of genotype *i*, *r*_*l*_ the effect of replicate *l*, *b*_*m*_ the effect of block *m* and ε_*ilm*_ the residual associated with plot for single trial analysis.

The variance component estimates from the mixed model analysis were used calculate the phenotypic (PCV) and genotypic (GCV) coefficients of variation as

(2) $$\begin{array}{r} PCV=\left(\frac{\sigma \text{p}}{\bar{X}}\right)100 \end{array}$$

(3) $$\begin{array}{r} GCV=\left(\frac{\sigma \text{g}}{\bar{X}}\right)100 \end{array}$$

where, σ_p_, σ_g_, and $\bar{X}$ were the phenotypic, genotypic standard deviation and grand mean of the traits, respectively (Singh and Chaudhary, 1985). Heritability in the broad sense (*H*^2^) was estimated on genotypic mean as:

(4) $$\begin{array}{r} H^{2}=\frac{\sigma^{2}g}{\sigma^{2}p} \end{array}$$

where σ^2^g and σ^2^p were genotypic and phenotypic variance, respectively (Allard, 1999; Galwey, 2014). Expected genetic advance (GA) and percentage of GA were calculated according to Shukla et al. (2006)

(5) $$\begin{array}{r} \text{GA}=\text{i}\sigma \text{p}{\text{H}}^{2} \end{array}$$

(6) $$\begin{array}{r} \text{GA}\left(\%\right)=\frac{\text{GA}}{\overset{-}{\text{X}}}\times 100 \end{array}$$

where *i* was standardized selection differential, a constant (2.08) and σ_p_ was the standard deviation of the phenotype.

A linear correlation analysis was applied in a pair-wise manner between traits to all the characteristics measured. SAS Proc MIXED model was employed for REML estimation of genetic and phenotypic correlations among traits and their standard errors following Holland (2006). The correlation matrixes between traits were visualized graphically with RCricos package (Krzywinski et al., 2009). Pairs of genetic and phenotypic correlation matrices from the REML estimation of each trial were used to calculate the overall magnitude of correlation within a matrix, average disparity and pattern of similarity between corresponding genetic and phenotypic correlation matrices. The overall magnitude of correlation within a matrix was calculated as:

(7) $$\begin{array}{r} \bar{x}=\frac{\sum |r_{i,j}|}{n},\text{ for }i\neq j, \end{array}$$

where *r*_*i,j*_ refers to the correlation between traits *i* and *j*, and *n* was the number of off-diagonal non-redundant elements in the matrix (Waitt and Levin, 1998).

The overall magnitude of correlation within a matrix was implemented to assess the extent to which phenotypic correlation reflected their genetic counterparts. The average disparity between corresponding genotypic and phenotypic correlation matrices was determined as:

(8) $$\begin{array}{r} D=\;\frac{\sum |r_{G,i,j}-r_{P,i,j}|}{n},\text{ for }i\neq j, \end{array}$$

where *r*_*G,i,j*_ and *r*_*P,i,j*_ referred to genetic and phenotypic correlations between trait *i* and *j*, and *n* was the number of off-diagonal non-redundant elements in the matrix (Willis et al., 1991). The average disparity between corresponding genetic and phenotypic correlation matrices was implemented to assess the proximity, on average, of the estimates of genotypic and phenotypic correlation. A pattern similarity of corresponding genetic and phenotypic correlation matrices was assessed using a matrix correlation based on:

(9) $$\begin{array}{r} r_{AB}=\frac{\sum_{i,j\;=\;1}^{n}\left(a_{ij}-\bar{a\;}ij\right)\left(b_{ij}-\bar{b\;}ij\right)}{\sqrt{\sum_{ij\;=\;1}^{n}{\left(a_{ij}-\bar{a\;}ij\right)}^{2}+\sum_{ij\;=\;1}^{n}{\left(b_{ij}-\bar{b\;}ij\right)}^{2}}},\text{ for }i\neq j \end{array}$$

where a_ij_ and b_ij_ were the i^th^ and j^th^ elements of correlation matrices being compared (A and B), $\bar{a\;}ij\;$ and $\bar{b\;}ij$ were mean of the off-diagonal elements in matrices A and B, respectively, and *n* was the number of traits sampled (cf. Waitt and Levin, 1998). The large positive matrix correlation indicated that genetic and phenotypic correlation vary in similar directions but not that the magnitudes of individual correlations were identical. Hence, the matrix correlation and average disparity were considered together for interpretation of the results. High matrix correlations and low average disparity indicated that the genetic and phenotypic correlation were similar in magnitude and tended to occupy the same position in their respective matrices (Waitt and Levin, 1998). The statistical significance of matrix correlation was tested using Mantel's randomization test (Mantel, 1967) in Genstat. In addition to assessing the magnitude, disparity and pattern similarity of corresponding genetic and phenotypic correlation matrixes, the causal relationship involved for the seed yield and all other component traits measured for the trials were assessed with path coefficient analysis as per the procedure described in Wright (1923, 1934). Level of genetic divergence of the diversity panels on DS attributes and their relative contribution to the differentiation of genotypes was assessed with Mahalanobis D^2^-statistics as described in Sharma (1998). The correlations analyses were done using SAS v 9.1software. Clustering and cluster statistics analyses were performed in R statistical software using various packages (eclodist, stats and clv). Mahalanobis's distance matrix in Ward2 hierarchical agglomerative clustering method (Murtagh and Legendre, 2014) was implemented to group the genotypes. The path coefficient analyses were performed using Microsoft Excel.

## Results

### Variation on seed yield and other traits

**Figure 1:**
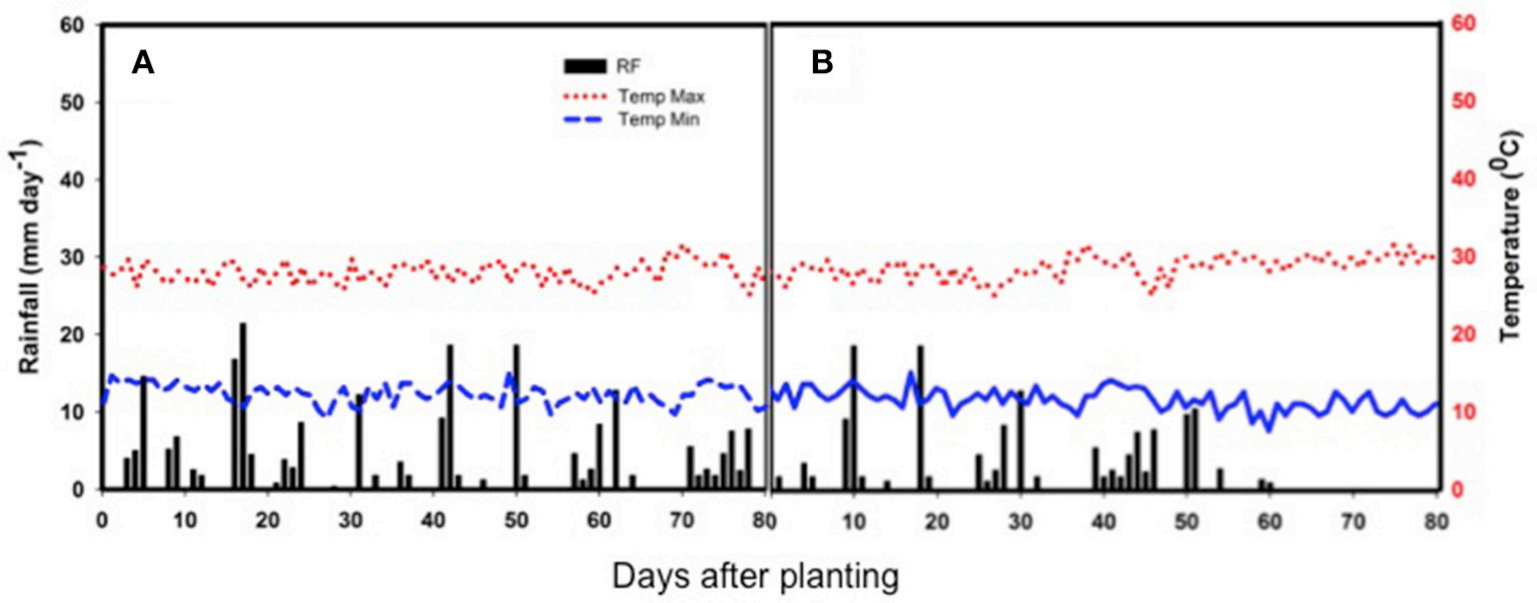
**Rainfall distribution (bars), as well as maximum and minimum temperatures (red and blue broken lines, respectively) during the crop growth period of two separately planted experiments (A)** non-stress with early sowing to avoid drought and **(B)** drought-stress due to late sowing to expose the crop to low overall rainfall and terminal drought. The x-axis shows days after planting at the bottom of the subfigures and the y-axes to the right and left of the graphs show the daily rainfall and temperature records, respectively.

The weather parameters (maximum and minimum daily temperatures and the daily total rainfall) during the crop-growing period are presented in Figure 1. The non-stress trial received a total rainfall of 235 mm during the growth period through evenly spaced rains (Figure 1A). Meanwhile the drought stress trial was planted later and only received 149 mm, with terminal drought (Figure 1B). The rainfall variation during the crop's life cycle caused a seed yield penalty under drought stress compared to the non-stress treatment and poor performance of plant traits in the terminal drought (Table 1).

**Table 1:** **Probability values for testing genotypic effect, mean traits values and REML estimates of variance components (σ^**2**^g, σ^**2**^e, σ^**2**^p), broad sense heritability (H^**2**^), genotypic (GCV) and phenotypic (PCV) coefficients of variation, genetic advance (GA%) and drought intensity index (DII%) for various adaptive and seed yield traits in Andean and Mesoamerican common bean gene pool diversity panels grown under contrasting water availability**.

**Trait**	**Gene pool**	**Stress level**	**Fprob**	**Mean**	**σ^2^g**	**σ^2^e**	**σ^2^p**	**H^2^**	**GCV%**	**PCV%**	**GA%**	**DII%**
YLDH	Andean	DS	<0.001	659	154871	14445	159686	0.97	59.71	60.63	122.31	59
		NS	<0.001	1619	230777	40075	244135	0.95	29.67	30.52	59.43	
	Meso	DS	<0.001	853	78018	24127	86060	0.91	32.74	34.38	64.83	63
		NS	<0.001	2315	317020	141292	364117	0.87	24.32	26.07	46.75	
DF	Andean	DS	<0.001	42	2.28	1.18	2.67	0.85	3.60	3.90	6.85	9
		NS	<0.001	46	9.60	2.23	10.34	0.93	6.78	7.03	13.45	
	Meso	DS	0.007	41	0.97	6.27	3.06	0.32	2.38	4.23	2.76	11
		NS	<0.001	46	10.78	1.86	11.40	0.95	7.18	7.38	14.38	
DPM	Andean	DS	<0.001	81	23.75	15.26	28.84	0.82	6.01	6.62	11.23	12
		NS	<0.001	92	8.88	4.89	10.51	0.84	3.23	3.52	6.12	
	Meso	DS	<0.001	77	4.59	13.58	9.12	0.50	2.78	3.91	4.06	16
		NS	<0.001	92	6.33	6.52	8.50	0.74	2.73	3.16	4.85	
SCMR	Andean	DS	<0.001	23.34	6.40	22.16	13.79	0.46	10.84	15.91	15.21	9
		NS	<0.001	25.63	14.53	17.09	20.23	0.72	14.87	17.55	25.97	
	Meso	DS	<0.001	20.88	11.48	6.57	13.67	0.84	16.23	17.71	30.63	17
		NS	<0.001	25.17	11.67	13.87	16.29	0.72	13.57	16.04	23.66	
PDPL	Andean	DS	<0.001	8.40	6.14	5.39	7.94	0.77	29.50	33.54	53.45	−39
		NS	0.006	6.06	0.51	2.41	1.31	0.39	11.79	18.90	15.14	
	Meso	DS	<0.001	10.19	8.35	4.09	9.71	0.86	28.35	30.58	54.16	29
		NS	0.006	14.30	9.48	22.62	17.02	0.56	21.53	28.85	33.10	
SDPD	Andean	DS	<0.001	3.05	0.38	0.24	0.46	0.82	20.17	22.21	37.72	37
		NS	<0.001	4.80	2.56	1.46	3.05	0.84	33.31	36.33	62.92	
	Meso	DS	<0.001	3.72	0.43	0.85	0.71	0.60	17.60	22.67	28.15	13
		NS	<0.001	4.27	0.38	1.48	0.88	0.44	14.51	21.94	19.77	
100SW	Andean	DS	<0.001	29.96	40.17	11.86	44.12	0.91	21.15	22.17	41.58	25
		NS	<0.001	39.79	64.49	17.48	70.32	0.92	20.18	21.07	39.82	
	Meso	DS	<0.001	18.58	19.29	5.65	21.17	0.91	23.64	24.77	46.48	27
		NS	<0.001	25.59	38.73	10.36	42.18	0.92	24.32	25.38	48.00	
PPI	Andean	DS	<0.001	45.05	279.20	128.00	321.87	0.87	37.09	39.82	71.16	13
		NS	<0.001	51.76	205.63	76.27	231.05	0.89	27.70	29.37	53.84	
	Meso	DS	<0.001	42.58	154.75	70.69	178.31	0.87	29.22	31.36	56.07	40
		NS	<0.001	71.31	290.10	131.30	333.87	0.87	23.88	25.62	45.86	
PHI	Andean	DS	<0.001	63.57	15.79	36.62	28.00	0.56	6.25	8.32	9.67	10
		NS	<0.001	70.81	16.87	15.93	22.18	0.76	5.80	6.65	10.42	
	Meso	DS	<0.001	68.59	19.77	25.15	28.15	0.70	6.48	7.74	11.19	10
		NS	<0.001	75.88	10.35	15.26	15.44	0.67	4.24	5.18	7.15	
HI	Andean	DS	<0.001	29.51	126.61	81.10	153.64	0.82	38.13	42.00	71.30	22
		NS	<0.001	37.63	118.28	66.33	140.39	0.84	28.90	31.49	54.65	
	Meso	DS	<0.001	29.51	79.33	50.15	96.05	0.83	30.18	33.21	56.51	46
		NS	<0.001	54.27	164.03	86.53	192.87	0.85	23.60	25.59	44.83	

*DF, days to flowering (number); DPM, days to harvest maturity (number); SCMR, SPAD leaf chlorophyll meter reading (SPAD); PDPL, pods per plant (number); SDPD, seeds per pod (number); 100SW, hundred seed weight (g); PPI, pod partitioning index (%); PHI, pod harvest index (%); HI, harvest index (%); DS, drought stress; NS, well-watered non-stress; Meso, Mesoamerican gene pool; Fprob, F probability to test genotypic difference. DII, drought intensity index presented as percentage reduction on trait expression compared with their nonstress counterpart*.

Drought stress resulted in a reduction in expression of all common bean traits assessed in this study except pods per plant in the Andean accessions in which a slight increase was observed with drought exposure. The overall seed yield reduction (drought severity index) was 59 and 63% under drought stress for the Andean and Mesoamerican gene pool accessions, respectively. Drought also caused early flowering and maturity in the bean diversity panels. Stress trials showed 9% (in Andean accessions) to 17% (in Mesoamerican accessions) reduction in leaf chlorophyll content. Exposure to drought also resulted in 37 and 13 % reduction in seeds per pod in Andean and Mesoamerican accessions, respectively. The stress on 100 seed weight and pod harvest index were generally mild irrespective of the gene pool origins as drought caused less than 30% reduction in these traits' mean performance in the accessions. Drought stress effects were lower for the Andean accessions in pod partitioning and harvest index compared to the Mesoamerican accessions as percent reduction in mean trait values were less than 30% Andean and above 40% in the Mesoamerican gene pool accessions.

In general, Mesoamerican diversity panel accessions showed slightly higher sensitivity to drought stress for expression of seed yield, days to flowering and maturity, pod partitioning index and harvest index compared to the Andean accessions for this environment. Andean accessions expressed more sensitivity to drought for expression of seed weight compared to the Mesoamerican accessions. Table 1 also presents different statistics for comparing the variation in various common bean plant traits between gene pools and across stress and non-stress conditions. Highly significant genotypic differences were observed for the expression of all the different traits assessed within the Andean and Mesoamerican gene pool accessions and across the drought stress and non-stress treatments.

The values of phenotypic coefficient variability (PCV) were higher than the corresponding genotypic coefficient of variability (GCV) values for all the traits within gene pools and across the water regimes. PCV values ranged from 3.90 to 60.63 % for Andean accessions and from 3.91 to 43.38 % for the Mesoamerican accessions under drought stress with the highest values for the trait of seed yield per hectare. Under non-stress treatment, the PCV values ranged from 3.52 (days to physiological to maturity) to 36.3 3% (seeds per pod) for the Andean gene pool accessions and 3.16 (days to physiological maturity) to 28.85% (pods per plant) for the Mesoamerican gene pool accessions.

Broad sense heritability estimates were in general greater than 0.50 for the majority of the traits except SCMR in the Andeans or DF and DPM in the Mesoamericans under drought stress, and PDPL in the Andeans or SDPD in the Mesoamericans under non-stress treatment. Genetic advance as percent of the mean was highest for yield (46.75–122.31%), 100 seed weight (39.82–48.00%) and pod partitioning index (45.86–71.16%) while the remaining traits showed a moderate to very low amounts of genetic advance over gene pools and across stress levels. The genetic advance values were observed to be higher for seed yield, pods per plant, pod partitioning index and harvest index on both gene pool accessions under drought stress compared to the non-stress treatment. This indicates that selection will be beneficial for improvement of these traits.

**Figure 2:**
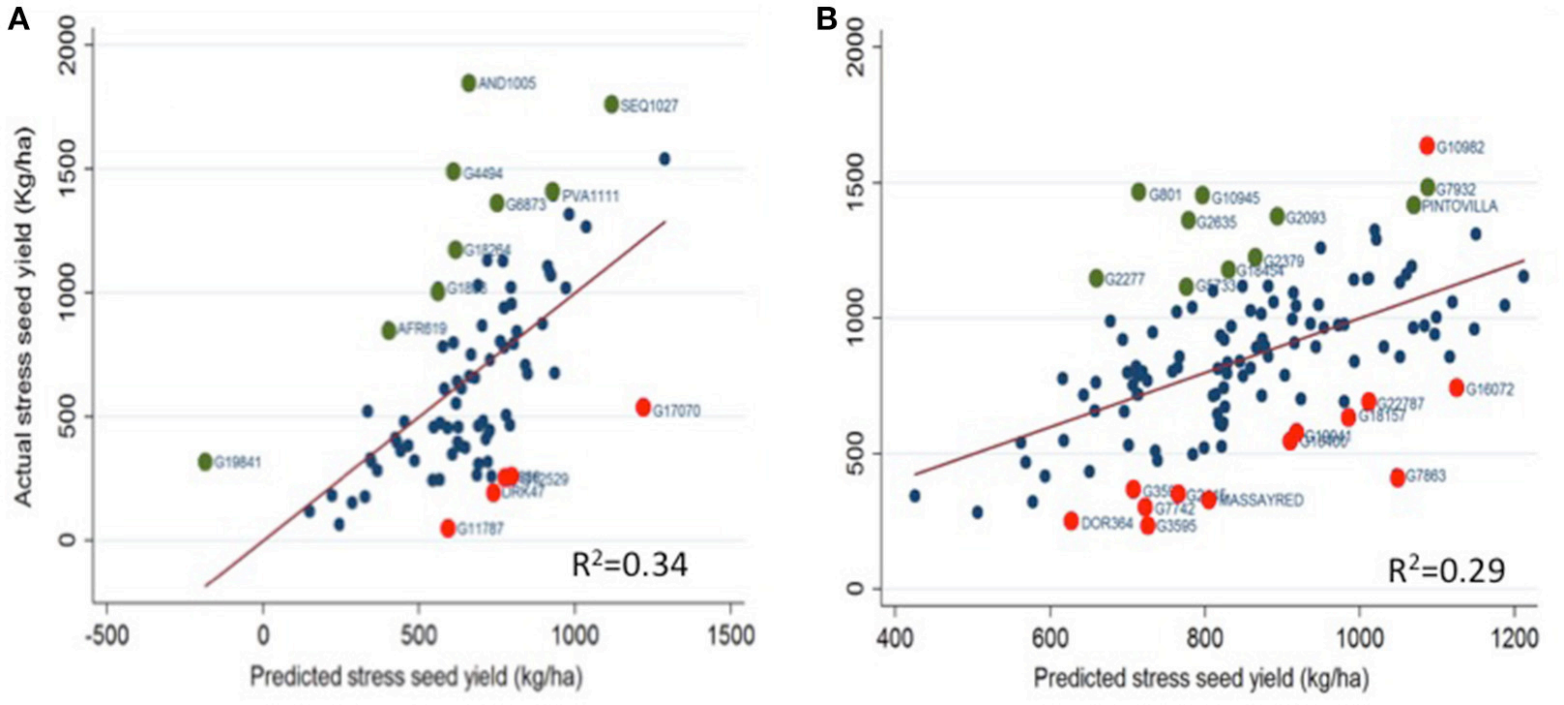
**Drought response of the common bean genotypes based on the residual variation in actual stress seed yield regressed on yield potential and growth duration and adjusted for experimental error**. The circles on the graph represent the genotype and deviation of the genotypes from the fitted regression line indicates the drought response. A genotype that expresses a high positive or negative deviation from the regression line could be defined as a drought resistance or susceptible, respectively. The green and red circles indicate genotypes with largest drought response indices hence are highly drought resistant and susceptible genotypes, respectively. **(A)** Andean genepool accessions, **(B)** Mesoamerican genepool accessions.

Figure 2 presents drought response of the genotypes in the trials based on the simultaneous regression of measured stress seed yield on the non-stress potential seed yield and growth duration (flowering and maturity time). The magnitude of deviation of a genotype from the regression line which is referred as a drought response index (DRI) was used to assess the response of genotypes to drought which is not explained by the yield potential and/or growth duration. We regressed measured stress seed yield on yield potential, growth duration (flowering and physiological maturity time) and DRI to assess the individual contribution of these four factors to explaining the variation in seed yield under stress. Yield potential and times to flowering were major factors contributing to variation in the seed yield in both gene pools. Yield potential explained an average of 23 and 15 % of the variation in seed yield under terminal drought in Andean and Mesoamerican accessions, respectively (data not presented). Flowering time explained an average of 11% of the variation in seed yield in both gene pools while times to physiological maturity was low in explaining the variation in seed yield under stress. Deviation from the regression or DRI explained 65% yield variation in Andean and 71% of the variation in seed yield under stress in Mesoamerican genepool accessions. More than half of the genotypes in the trial (both Andean and Mesoamerican genepool) expressed no specific response to the drought stress indicating their measured seed yield under stress was adequately estimated by their potential yield and growth duration (flowering time and maturity). These genotypes are those with a drought response index value of near zero or within the limits of experimental error hence are average in their susceptibility to drought. Few genotypes expressed a high deviation from the regression line or high DRI values. Genotypes such as AND1005, SEQ1027, PVA111, G6873, G4493, G1836, and AFR619 from the Andean genepool and G801, G10945, G10982, G18454, G2093, G2277, G2379, G2635, G5733, G7932, and PINTOVILLA from the Mesoamerican gene pool with a high and positive deviation from the regression line showed a resistance reaction to the drought stress treatment. Whereas, G2686, G17070, G12529, G11787, and DRK47 from the Andean accessions and DOR364, MASSAYRED, G16400, G1815, G19941, G22787, G2445, G3595, G7742, and G7863 from the Mesoamerican accessions with a negative and higher deviations from the regression line expressed a susceptible response to drought. Application of the drought response index in this study identified drought resistant and susceptible genotypes of common beans. Such an attempt to dissect the genotype's drought response status should consider also the absolute yield under non-stress and drought stress along with other traits defining drought adaptation and cultivar acceptability in breeding of common beans for tropical environments.

### Correlations among adaptive traits and between the seed yield

**Figure 3:**
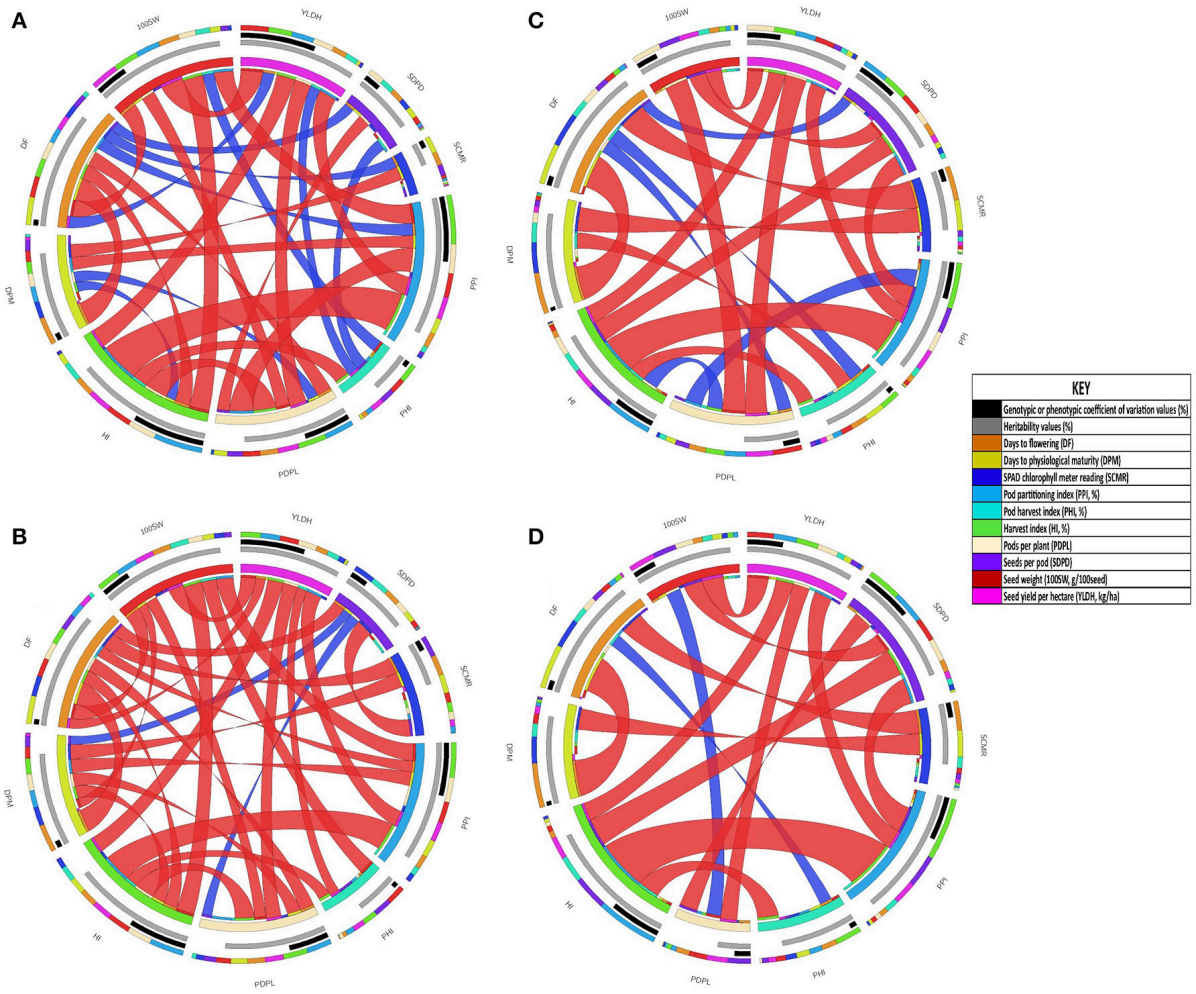
**Graphical display of the correlation matrices, variance coefficients and heritability of traits in Andean gene pool diversity panels grown under drought stress and non-stress treatments at Hawassa, Ethiopia, in 2009. (A)** phenotypic correlation under drought stress, **(B)** genotypic correlations under drought stress, **(C)** phenotypic correlation under non-stress and **(D)** genotypic correlations under non-stress. The color specifications in the subfigures: the outer circle is the correlation coefficient values of a trait with others represented in different colors (all traits have separate colors as indicated in Key) and sizes irrespective of its direction, the middle circles black represent the sizes of genotypic or phenotypic coefficient of variation values and gray represent sizes of heritability values which proportional to the length of circle segment (for the detail values refer table 1). The ribbons in the inner circle represent significant correlations red (significant at 0.01 p value) and blue (significant at 0.05 *p* value). Ribbons are twisted if the correlation is negative and flat if positive (detail correlation coefficient values presented in Supplementary Table 1).

Correlations between seed yield and adaptive traits determined for accessions of the two common bean gene pools evaluated under both drought stress and non-stress treatments are presented in Figure 3 and Table 2. Seed yield had positive and high genotypic and phenotypic correlation with PDPL, 100SW, PPI, and HI under both drought stress and non-stress treatments in Andean gene pool accessions (Figure 3, Supplementary Table 1). While for the Mesoamerican gene pool accessions, the correlation with seed yield was positive and significant only for PDPL both at genotypic and phenotypic levels across the water regimes (Table 2). Similarly, days to flowering had a significant and positive genotypic and phenotypic correlation with days to physiological maturity over the gene pools and across the water regimes. SPAD chlorophyll meter reading had significant but negative genotypic and phenotypic correlation with days to flowering and days to physiological maturity over the gene pool and across water regimes. The association of SAPD chlorophyll reading with SDPD was significant but negative both at genotypic and phenotypic correlation under drought stress while it was significant and negative at phenotypic level and non-significant genotypic level under non-stress treatment for the Andean accessions. Correlations among yield parameters were more complex than for the phenological and photosynthetic traits. For example, the correlation between PDPL and SDPD was negligible both at genotypic and phenotypic levels across the drought stress and non-stress treatments for the Mesoamerican accessions, but was significant at 0.05 *p* value for the Andean accessions. Hundred seed weight had negative and highly significant genotypic and phenotypic correlations at 0.01 *p* value with days to flowering under drought stress in both gene pool accessions.

**Table 2:** **Estimates of correlation coefficients between of common bean traits (genetic below diagonal and phenotypic above diagonal) in Mesoamerican gene pool diversity panels grown under drought stress (DS) and non-stress (NS) treatments at Hawassa, Ethiopia, in 2009**.

	**Stress regime**	**YLDH**	**DF**	**DPM**	**SCMR**	**PDPL**	**SDPD**	**100SW**	**PPI**	**PHI**	**HI**
YLDH	DS	1.00	−0.16	−0.18	0.15	0.28^**^	0.04	0.30^**^	0.27^**^	0.19^*^	0.26^**^
	NS	1.00	0.20^*^	0.28^**^	−0.19	0.26^**^	0.07	−0.12	0.14	−0.03	0.12
DF	DS	−0.44^*^	1.00	0.41^**^	−0.08	0.0001	0.08	−0.20^*^	−0.12	0.04	−0.09
	NS	0.29^**^	1.00	0.58^**^	−0.17	0.21^*^	0.11	−0.47^**^	0.05	−0.33^**^	−0.05
DPM	DS	−0.32^**^	0.62^**^	1.00	−0.05	−0.02	0.07	−0.12	−0.20^*^	−0.03	−0.16
	NS	0.29^*^	0.81^**^	1.00	−0.12	0.25^**^	−0.04	−0.17	0.12	−0.30^**^	0.02
SCMR	DS	0.23^*^	−0.38	−0.20	1.00	0.02	−0.18^*^	0.46^**^	0.04	0.16	0.07
	NS	−0.23^*^	−0.30^**^	−0.21	1.00	−0.10	−0.12	0.25^*^	0.06	0.16	0.09
PDPL	DS	0.41^**^	0.004	−0.06	0.04	1.00	−0.07	0.01	0.35^**^	0.01	0.32^**^
	NS	0.48^**^	0.47^**^	0.54^**^	−0.13	1.00	−0.17	−0.22^*^	0.35^**^	−0.17	0.30^**^
SDPD	DS	0.09	0.52	0.17	−0.26^*^	0.07	1.00	−0.30^**^	0.19^*^	0.24^*^	0.28^**^
	NS	0.28	0.25	−0.12	−0.20	0.39	1.00	−0.30^**^	0.13	0.06	0.16
100SW	DS	0.33^**^	−0.62^*^	−0.28^*^	0.65^**^	0.03	−0.53^**^	1.00	0.17	0.20^*^	0.17
	NS	−0.21^*^	−0.57^**^	−0.31^**^	0.48^**^	−0.50^**^	−0.64^**^	1.00	−0.08	0.34^**^	−0.003
PPI	DS	0.36^**^	−0.52^*^	−0.56^**^	0.08	0.49^**^	0.30^*^	0.23^*^	1.00	0.14	0.91^**^
	NS	0.18^**^	0.07	0.27^**^	0.07	0.46^**^	0.44^**^	−0.11^*^	1.00	−0.04	0.95^**^
PHI	DS	0.30^**^	0.19	−0.09	0.35^**^	0.10	0.12	0.35^**^	0.24^*^	1.00	0.32^**^
	NS	−0.14	−0.56^**^	−0.54^**^	0.51^**^	−0.28	−0.04	0.60^**^	0.03	1.00	0.17
HI	DS	0.37^**^	−0.42	−0.53^**^	0.13	0.48^**^	0.33^**^	0.24^*^	0.98^**^	0.39^**^	1.00
	NS	0.15^**^	−0.05	0.15	0.14	0.40^**^	0.45	−0.02	0.99^**^	0.17	1.00

*, ** *, Significant at 5 and 1% probability levels, respectively. DF, days to flowering (number); DPM, days to harvest maturity (number); SCMR, SPAD leaf chlorophyll meter reading (SPAD); PDPL, pods per plant (number); SDPD, seeds per pod (number); 100SW, hundred seed weight (g); PPI, pod partitioning index (%); PHI, pod harvest index (%); HI, harvest index (%)*.

Under the non-stress treatment, 100 seed weight showed negative and significant correlation with days to flowering both at genotypic and phenotypic level for the Mesoamerican accessions while it was negative and high at phenotypic level and weak at the genotypic level for the Andean accessions. Pod partitioning index showed strong positive correlation (both at genetic and phenotypic level) with harvest index over the gene pools and across stress factors. The positive and significant associations were observed between the two indices HI and PHI, both at genotypic and phenotypic levels under the drought stress on both gene pool accessions while the same correlation was positive and significant at genotypic and phenotypic levels for the Andean accessions and positive but non-significant for the Mesoamerican accessions under non-stress. The correlations of pods per plant with seed yield, harvest index with pod harvest index, and days to flowering with days to physiological maturity suggest the potential for simultaneous improvement of these traits through breeding. Furthermore, selections for increased levels of traits such as pods per plant, seed weight, pod partitioning index and harvest index in Andean and pods per plant in Mesoamerican gene pool accessions would improve the seed yielding potential under both drought stress and non-stress environments.

### Magnitude, disparity and pattern similarity of genetic and phenotypic correlations

The corresponding pairs of genetic and phenotypic correlations of traits in the diversity panels representing the Andean and Mesoamerican gene pools of common bean grown under contrasting water regimes (well-watered non-stress and water stressed terminal drought treatments) were compared with average absolute values of genetic and phenotypic correlation matrices, average disparity and matrix correlation (Table 3). The overall magnitude of correlations as measured by absolute value had a mean value of 0.313 for the genotypic correlation compared with 0.206 for the corresponding phenotypic correlation.

**Table 3:** **Comparison of the corresponding pairs of genetic and phenotypic correlations common bean traits grown under contrasting water regimes, where /RG/ and /Rp/ are the average absolute values of genetic and phenotypic correlation, respectively; D is the average disparity; rM is the matrix correlation between genetic and phenotypic correlation matrices; PrM is the proportion of permutation matrix correlation exceeding the observed matrix correction**.

**Gene pool**	**Stress regime**	**Correlation with and among traits**	**No traits**	**No corr**	**/RG/**	**/Rp/**	**D**	**rM**	**PrM**
Andean	NS	Between yield and others	9	9	0.272	0.194	0.080	0.954	NA
		Among all traits	10	45	0.290	0.200	0.107	0.956	0.01
	DS	Between yield and others	9	9	0.346	0.267	0.280	0.982	NA
		Among all traits	10	45	0.385	0.256	0.134	0.974	0.01
	NS vs. DS	r_g,i,j_ at DS with corresponding r_g,i,j_ at NS	10	45	NA	NA	0.247	0.757	0.01
		r_p,i,j_ at DS with corresponding r_p,i,j_ at NS	10	45	NA	NA	0.181	0.706	0.01
		r_g,i,j_ at DS with corresponding r_p,i,j_ at NS	10	45	NA	NA	0.282	0.672	0.01
		r_p,i,j_ DS with r_g,i,j_ NS of all traits	10	45	NA	NA	0.182	0.767	0.01
Mesoamerican	NS	Between yield and others	9	9	0.250	0.157	0.093	0.974	NA
		Among all traits	10	45	0.323	0.192	0.143	0.936	0.01
	DS	Between yield and others	9	9	0.317	0.203	0.114	0.944	NA
		Among all traits	10	45	0.320	0.180	0.148	0.918	0.01
	NS vs. DS	r_g,i,j_ at DS with corresponding r_p,i,j_ at NS	10	45	NA	NA	0.275	0.524	0.70
		r_p,i,j_ at DS with corresponding r_p,i,j_ at NS	10	45	NA	NA	0.151	0.675	0.01
		r_g,i,j_ at DS with corresponding r_p,i,j_ at NS	10	45	NA	NA	0.246	0.569	0.01
		r_p,i,j_ DS with r_g,i,j_ NS of all traits	10	45	NA	NA	0.262	0.563	0.10

*NS, well watered non-stress; DS, drought stress; r_g,i,j_, genetic correlation between trait i and j; r_p,i,j_, phenotypic correlation between trait i and j; No corr, number of correlation; NA, not applicable. PrM is the probability of obtaining a matrix correlation as high or higher by chance. If PrM is less than the table wide significance level of 0.01, then the null hypothesis of no pattern similarity is rejected*.

The overall magnitude of genetic correlation matrices was higher than the corresponding phenotypic correlation in 100% of the comparisons (Table 3). The average disparity as a measure of how close, on average, the corresponding correlation matrices was ranged from a low of 0.080 to a high of 0.282. The correlations of seed yield with other traits in Andean accessions under non-stress had little disparity whereas genetic correlation among all traits in Andean accessions evaluated under drought stress with its corresponding phenotypic correlation matrices under non-stress showed a high disparity.

The matrix correlation that measured the pattern similarity of the magnitude of the corresponding correlation matrices ranged from 0.524 to 0.982 with a mean value of 0.804 (Table 3). The phenotypic correlation in the Mesoamerican accessions under drought stress with the corresponding phenotypic correlation under non-stress showed little pattern similarity while the corresponding genotypic and phenotypic correlation between seed yield and other traits under drought stress in Andean accessions showed high pattern similarity. Eighty-three percent of the corresponding matrix correlations were more similar than would be anticipated by chance alone with the table-wide significance level of 0.01.

### Causal relationship of seed yield with other traits

Path coefficients between individual traits and YLDH were calculated separately for the Andean and Mesoamerican gene pool accessions and were useful for dissection of the inter-relationships between the adaptive traits and their effects on seed yield at the genotypic level based on the correlation matrices (with direct effects on the main diagonal and indirect effects on both off-diagonal parts of the table). The genotypic correlation matrices of adaptive traits with the seed yield in each gene pool were asymmetrical, consisting of nine components. Meanwhile the direct genotypic effect and the indirect genotypic effects of adaptive traits on seed yield had relationships with the other eight traits.

For the Andean gene pool accessions (Table 4), the direct genotypic effects of pods per plant and seeds per pod on seed yield were positive, and those of SPAD leaf chlorophyll meter reading and pod partitioning index were negative over the stress factors. Days to flowering, 100 seed weight and harvest index had positive and negative direct genotypic effect on seed yield under drought stress and non-stress, respectively. Days to physiological maturity and pod partitioning index had negative direct genotypic effects on seed yield under drought stress and positive direct genotypic effects under non-stress treatment. Days to physiological maturity and pod harvest index had direct positive impact on seed yield under non-stress and negative direct effect under drought stress. The largest direct positive effect was that of harvest index (3.06) under drought stress, days to physiological maturity (1.46) and pods per plant (1.21) under non-stress. The indirect genotypic effect of pods per plant, 100 seed weight, pod portioning index, and pod harvest index via harvest index on seed yield were high and positive under drought stress while the same paths had a negative indirect effect on seed yield under non-stress in the Andean gene pool accessions.

**Table 4:** **Direct (Diagonal) and indirect (off-diagonal) genotypic effect path coefficient of adaptive traits on seed yield (variable YLDH, abbreviated as Y) on Andean common bean gene pool accessions evaluated under drought stress and non-stress environments**.

**Trait**	**Stress regime**		**Path coefficient (P**_**i→j→y**_**)**								**r_g with YLDH_**
		**DF→Y**	**DPM→Y**	**SCMR→Y**	**PDPL→Y**	**SDPD→Y**	**100SW→Y**	**PPI→Y**	**PHI→Y**	**HI→Y**	
DF→	DS	0.52	−0.20	0.09	−0.11	0.06	−0.38	1.05	0.07	−1.44	−0.34
	NS	−1.21	1.28	0.04	−0.44	0.27	0.06	0.03	−0.14	0.09	−0.02
DPM→	DS	0.41	−0.26	0.08	−0.10	0.04	−0.24	1.33	0.01	−1.35	−0.08
	NS	−1.07	1.46	0.03	−0.27	0.12	−0.02	0.01	−0.16	0.02	0.13
SCMR→	DS	−0.30	0.14	−0.15	0.05	−0.08	0.20	−0.50	0.01	0.83	0.19
	NS	0.89	−0.95	−0.05	0.07	−0.12	0.02	0.00	0.04	0.02	−0.09
PDPL→	DS	−0.25	0.12	−0.03	0.22	−0.05	0.30	−1.94	0.07	2.08	0.52
	NS	0.44	−0.32	0.00	1.21	−0.29	−0.17	−0.09	0.06	−0.25	0.58
SDPD→	DS	0.23	−0.08	0.09	−0.08	0.14	−0.16	0.11	−0.33	0.06	−0.01
	NS	−0.34	0.19	0.01	−0.36	0.96	0.12	−0.11	0.04	−0.34	0.17
100SW→	DS	−0.27	0.08	−0.04	0.09	−0.03	0.74	−1.66	−0.34	1.96	0.53
	NS	0.25	0.13	0.00	0.75	−0.43	−0.28	−0.02	0.09	−0.09	0.42
PPI→	DS	−0.20	0.12	−0.03	0.15	−0.01	0.44	−2.77	−0.15	2.97	0.54
	NS	0.18	−0.06	0.00	0.55	0.53	−0.03	−0.20	0.08	−0.60	0.44
PHI→	DS	−0.06	0.00	0.00	−0.02	0.07	0.38	−0.64	−0.66	1.25	0.34
	NS	0.45	−0.61	−0.01	0.18	0.11	−0.07	−0.04	0.39	−0.26	0.14
HI→	DS	−0.24	0.11	−0.04	0.15	0.00	0.47	−2.69	−0.27	3.06	0.56
	NS	0.18	−0.06	0.00	0.50	0.53	−0.04	−0.20	0.16	−0.62	0.46

*DS, drought stress; NS, well-watered non-stress; Y, represents YLDH; r_g_, genotypic correlation with seed yield per hectare. For trait abbreviation refer Table 1*.

For the Mesoamerican gene pools accessions (Table 5), days to physiological maturity and 100 seed weight had a positive direct effect, and pod harvest index had a negative direct effect on seed yield over stress factor. Days to flowering, SPAD leaf chlorophyll meter reading, pod partitioning index and harvest index had positive direct genetic effects on seed yield under drought stress and negative under non-stress. Pods per plant, seeds per pod and harvest index had negative direct genetic effects on seed yield under drought stress and highest positive direct genetic effect under non-stress. Harvest index had positive indirect under non-stress. Under drought stress, pod-partitioning index had the highest positive direct genetic effect on seed yield.

**Table 5:** **Direct (Diagonal) and indirect (off-diagonal) genotypic effect path coefficient of adaptive traits on seed yield on Mesoamerican common bean gene pool accessions evaluated under drought stress and non-stress environments**.

**Trait**	**Stress regime**	**Path coefficient (P**_**i→j→y**_**)**									**r_g with YLDH_**
		**DF→Y**	**DPM→Y**	**SCMR→Y**	**PDPL→Y**	**SDPD→Y**	**100SW→Y**	**PPI→Y**	**PHI→Y**	**HI→Y**	
DF→	DS	4.46	0.01	−0.54	−0.01	−1.34	−0.12	−5.77	−0.10	2.98	−0.44
	NS	−0.27	0.62	0.06	0.20	0.17	−0.30	−0.24	0.20	−0.16	0.29
DPM→	DS	2.76	0.02	−0.28	0.09	−0.44	−0.05	−6.22	0.05	3.76	−0.32
	NS	−0.22	0.76	0.05	0.23	−0.08	−0.16	−0.94	0.20	0.47	0.29
SCMR→	DS	−1.69	0.00	1.41	−0.06	0.67	0.13	0.89	−0.19	−0.92	0.23
	NS	0.08	−0.16	−0.21	−0.06	−0.14	0.26	−0.24	−0.18	0.43	−0.23
PDPL→	DS	0.02	0.00	0.06	−1.47	−0.18	0.01	5.44	−0.05	−3.40	0.41
	NS	−0.13	0.41	0.03	0.43	0.27	−0.27	−1.61	0.10	1.24	0.48
SDPD→	DS	2.32	0.00	−0.37	−0.10	−2.58	−0.10	3.33	−0.07	−2.34	0.09
	NS	−0.07	−0.09	0.04	0.17	0.70	−0.34	−1.54	0.01	1.40	0.28
100SW→	DS	−2.76	0.00	0.92	−0.04	1.37	0.19	2.55	−0.19	−1.70	0.33
	NS	0.15	−0.24	−0.10	−0.21	−0.45	0.53	0.38	−0.22	−0.06	−0.21
PPI→	DS	−2.32	−0.01	0.11	−0.72	−0.78	0.04	11.10	−0.13	−6.94	0.36
	NS	−0.02	0.21	−0.02	0.20	0.31	−0.06	−3.50	−0.01	3.07	0.18
PHI→	DS	0.85	0.00	0.49	−0.15	−0.31	0.07	2.66	−0.55	−2.76	0.30
	NS	0.15	−0.41	−0.11	−0.12	−0.03	0.32	−0.10	−0.36	0.53	−0.14
HI→	DS	−1.87	−0.01	0.18	−0.71	−0.85	0.05	10.88	−0.21	−7.09	0.37
	NS	0.01	0.11	−0.03	0.17	0.31	−0.01	−3.46	−0.06	3.10	0.15

*DS, drought stress; NS, well-watered non-stress; Y, represents YLDH; r_g_, genotypic correlation with seed yield per hectare. For trait abbreviations refer Table 1*.

Meanwhile for this same gene pool, harvest index had genetic effects on seed yield through SPAD leaf chlorophyll meter reading, pods per plant, seeds per pod, pod partitioning index, and pod harvest index under non-stress whereas the same route had negative indirect genetic effect on seed yield under drought stress. Harvest index had the larger positive indirect genetic effect on seed yield through pod partitioning index under drought stress in the Mesoamerican gene pool accessions.

The path analysis results in Tables 4, 5 depicted different trait responses involve in modulating the seed yielding potential of common bean plants in the two gene pools. Pods per plant and seeds per pod in Andean accessions and seed weight (grain filling) in Mesoamerican accessions are potential traits to practice indirect selection improving seed yield both under drought stressed and non-stressed environments. In addition, harvest index, seed weight (better grain filling) and flowering time in Andean accessions and pod partitioning index, leaf chlorophyll content, and flowering time in Mesoamerican accessions are relevant traits to attempt indirect selection improving the seed yielding potential under drought stress.

### Differentiation on the basis of phenotypic performance

**Figure 4:**
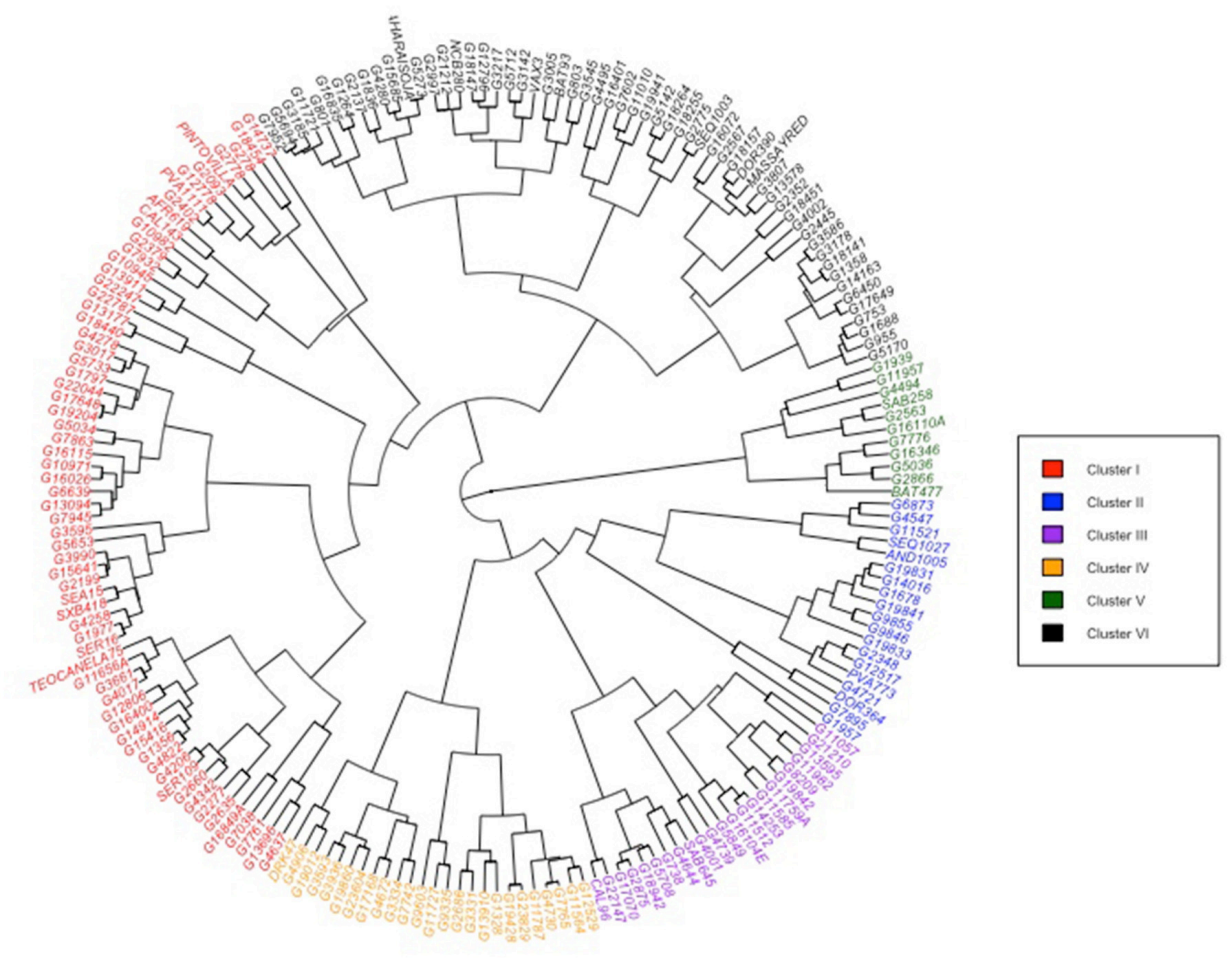
**Phenogram showing hierarchical grouping patterns of 202 common bean accession in two diversity panels (Andean and Mesoamerican) and grouped in six clusters based on reaction to drought-stress condition**. Mahalanobis's generalized distance used for clustering based on ten quantitative trait variables measured across all genotypes.

Clustering analysis of the phenotypic performance under drought stress of the total number of 202 accessions representing the two common bean gene pools using the Mahalanobis's distance in Ward's hierarchical agglomerative clustering method grouped the genotypes in six clusters (Figure 4). The clustering analysis with phenotypic traits did not generate a meaningful differentiation of the accessions into Andean and Mesoamerican gene pools as well as the distinctness in degree of drought adaptation. Accessions from the two gene pools and with varied of level of drought tolerance tended to cluster together. This indicates different mechanisms of plant responses to drought adaptation involve in common bean. The Andean accessions with a high degree of drought tolerance such as CAL143, G19882, AND1005, and SEQ1027 were grouped under clusters I and II while those from the Mesoamerican gene pools such as G10982, G7932, G10945, and PINTOVILLA were grouped under cluster I. Accessions G10982, G10945, and G2402 which belongs to the race Durango of highland Mexico were grouped with the drought bred lines SER16, SEA15, SXB418, and SER109 in cluster I. The genotypes in cluster I will be good sources of genes for drought adaptation in future breeding. Cluster size varied between the groups identified: with a larger number of genotypes in cluster I (33%) and cluster VI (29% of genotypes); whereas clusters V and II were small groups containing 11 (5%) and 19 genotypes (9%), respectively. Clusters III and V together had 24% of all the genotypes.

**Figure 5:**
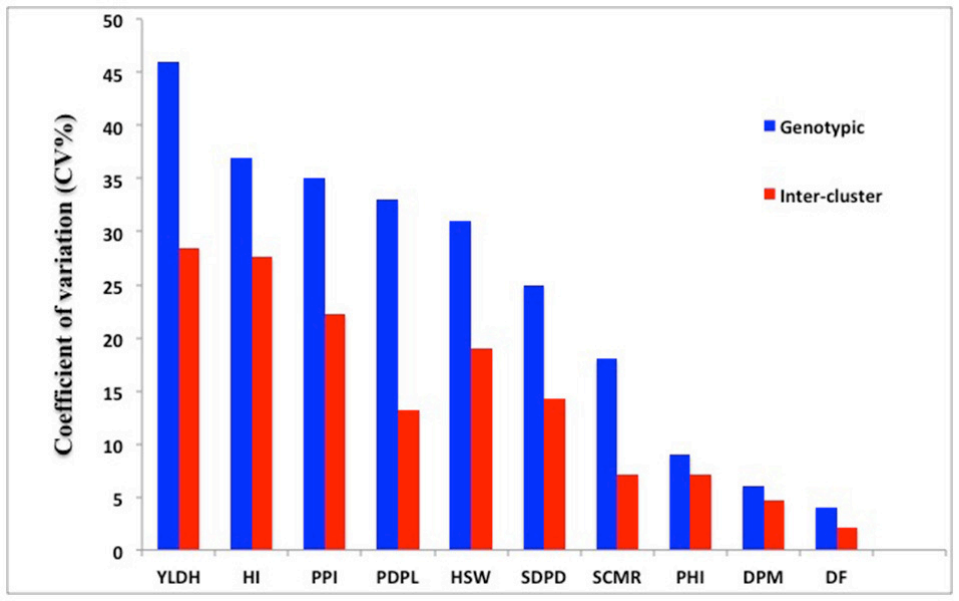
**The relative contribution of the multiple traits to the phenotypic differentiation in common bean diversity panels evaluated under drought stress at a genotypic and inter-cluster level as demonstrated by coefficient of variation (CV%) values**. Y-axis is CV value for the trait and x-axis is multiple traits contributing to the group formation. Refer Table 1 for trait abbreviations.

The Mahalanobis generalized distance for the 202 genotypes ranged from 0.46 to 97.52. The highest inter-cluster divergence appeared between clusters II and V (*D*^2^ = 30.89) followed by clusters III and V (*D*^2^ = 39.10) (Table 6). The lowest inter-cluster distance was found between clusters I and VI (*D*^2^ = 17.74) followed by that between clusters III and VI (*D*^2^ = 18.78) and IV and VI. The relative contribution of the multiple traits to the differentiation of the diversity panels at genotypic and the inter-cluster level is demonstrated by the coefficient of variation (CV) values (Figure 5). Seed yield, harvest index, pod partitioning index and 100 seed weight are potential contributors to phenotypic differentiation both at the genotype and inter-cluster level in the common bean gene pool accessions considered for this study. Days to flowering, days to maturity and pod partitioning index were least contributors to phenotypic divergence in the reference collections. Seeds per pod and leaf chlorophyll content were intermediate in contribution to the differentiation of accessions. This indicates no trait is equally important to phenotypic differentiation and drought adaptation in common beans. Methods to recognize relevant traits to drought adaptation in common beans should consider the relevance of different traits in different environmental situations.

**Table 6:** **Intra (diagonal values) and inter-cluster divergence (off-diagonal values) in 202 common bean diversity panels evaluated under drought stress based on Mahalanobis's generalized distance**.

**Clusters**	**I**	**II**	**III**	**IV**	**V**	**VI**
I (66)	**8.78**	25.10	20.71	22.24	27.99	17.74
II (19)		**10.35**	23.98	24.27	30.89	22.34
III (23)			**7.20**	20.87	29.10	18.78
IV (25)				**6.95**	27.23	18.75
V (11)					**8.29**	24.22
VI (58)						**6.24**
χ2_0.01_ = 20.09						
Significant at *P* < 0.01						

*Values in parenthesis are number of accessions forming cluster (group of accessions resembling each other for their phenotypic performance under drought stress for the ten traits and hence with low intra-cluster divergence). Bold values refer to intra-cluster distance. The χ2 significance was calculated using CHIINV functions in Microsoft excel with n-2 degree of freedom, where n is the number of traits considered for clustering which in this case were 10 traits*.

## Discussion

The drought stress imposed by deficient water availability during the post-rainy season had a significant impact on the seed yield potential, overall performance and plant attributes of the genotypes. The total rainfall received during the drought stress treatment was 37% lower than that of the non-stress treatment. The drought experiment received a low amount of rainfall during flowering that coupled with a total cut-off of rain during pod filling exposed the common bean plant to water limitation over a long critical growth period for the plants (Figure 1). In contrast, the amount and distribution of rainfall over the growth period for the non-stress, rainy-season trial, was near to the average rainfall required by the common bean crop for the optimum growth and development (350–500 mm). In summary, the pattern of rainfall in the Ethiopian highlands allows for drought tolerance testing using staggered alternate sowing dates as shown by our results.

The degree of stress on seed yield potential imposed by alternating the sowing dates as expressed by drought intensity index values were 59 and 63% for the Andean and Mesoamerican gene pool panel accessions, respectively. This drought level was high and adequate to reveal genotypic differences, as seen by the differential response of the genotypes for the various traits measured (Table 1). Previous research on common bean also successfully created consistent stress between experiments by alternating sowing dates to expose the crop to high and low rainfall during the growing period (Acosta-Gallego and White, 1995; Abebe et al., 1998; Asfaw et al., 2012; Rezene et al., 2013; Asfaw and Blair, 2014). Due to the environment used, the kind of abiotic stress that the common bean plants experienced under this study was terminal drought stress, which is a serious problem for common bean production in the tropics (Rao et al., 2013; Beebe et al., 2014). This study reflected the typical stress condition that common bean growers often face in Ethiopia and also was in accordance with other drought studies in tropical regions where the crop is often grown during the short rains, when precipitation totals decline early (Rao et al., 2013; Ambachew et al., 2015; Darkwa et al., 2016).

Persistent or intermittent water limitation at any time during the growing season poses a great challenge for common bean production worldwide (Terán and Singh, 2002). This problem is further exacerbated in the rain-fed common bean production system in the tropics where the application of irrigation water is not very feasible, due to deep-rooted poverty and lack of infrastructure. Use of climate resilient varieties is among series of action steps to mitigate the drought problem and position the common bean crop to have a greater role for the livelihood improvement in drought prone production systems of the tropics.

Our study assessed the dynamics of trait linkage and the variability in two major common bean gene pool reference diversity panels grown under contrasting water availability, which provided additional insights instrumental for the making of new resilient varieties. The high variability for and strong positive associations among some adaptive traits over the gene pools and across stress levels were critical aspects of our study which could be useful to common bean breeding programs in best parent selection and trait targets necessary for progress in drought adaptation. The magnitude of drought resistance index for seed yield highlighted the response of some accessions over others (Figure 2) but our results also indicated that drought tolerance was not a function of a single plant trait but rather a cumulative function of many interdependent traits, as observed before (Rao, 2001; Asfaw et al., 2012; Beebe et al., 2013). Traits like pods per plant, pod partitioning index and harvest index showed high heritability combined with good genetic advance across both gene pools and under both treatments hence are important for crop improvement under drought stress. Understanding the pattern of association of such traits at genotypic and phenotypic level is of practical interest in a breeding program as selection for one trait may cause either improvement or deterioration in an associated trait (Baker, 1986; Rao et al., 2013; Ambachew et al., 2015).

In addition to increasing seeds and pods per plant for higher yield, seed weight (grain filling), pod partitioning index and harvest index can be used to improve seed yield for both drought stress and non-stress conditions in Andean gene pool accessions. However, for the Mesoamerican gene pool accessions, increases in pods per plant were associated with seed yield only under non-stress conditions while many pods were unfilled in drought stress. Hence, pods per plant, seed weight, pod partitioning index and harvest index are useful selection objectives to improve drought adaptation in common bean, but must be differentially weighted in each gene pool.

The extent to which the phenotypic correlations between traits reflect its genotypic counterpart is another aspect a breeding program has to take into account while designing a selection strategy for the crop. In this study, the correlation analyses for multiple traits indicated the higher values for genetic correlation matrices compared to the corresponding phenotypic matrices for all the traits, gene pools and stress levels. A review on genotypic and phenotypic correlations in 27 different species revealed that phenotypic correlation is a good reflection of genotypic correlations in plants (Waitt and Levin, 1998). As previously reported by Asfaw and Blair (2014) and Ambachew et al. (2015), the greater contribution of genotypic factors in the development of trait linkages and the strong positive correction between phenotypic traits may be an indication of pleiotropism, genetic coupling and / or linkage disequilibrium with population structure effects. In any case these genetic phenomena provide the opportunity to select superior genotypes that indeed offer increased seed yield and other desirable traits simultaneously (Dilday et al., 1990; Sharma, 1998).

Drought tolerance in common bean is made up of multiple sub-traits and is always quantitative in the nature of its inheritance. Considering trait association analysis results along with genetic diversity assessment could assist bean breeders to pyramid the different plant traits linked with drought tolerance. This study dissected level of difference among study materials for the plant attributes on drought stress treatment. The genetic differentiation assessment with Mahanalobis *D*^2^ statistics indicated a high degree of phenotypic divergence among common bean accessions, between genepools and therefore within and between the reference Andean and Mesoamerican diversity panels. Genotype classification with phenotypic traits did not reveal clear distinction of the least and most highly drought-adapted genotypes belonging to different groups or genepools. This may be explained by the complementarity of drought tolerance traits found crossing the genepool divisions of Andean and Mesoamerican common beans. Accessions from different gene pools clustered together irrespective of their degree of phenotypic resemblance for drought response traits measured in this study and their genepool status, allowing us to conclude that drought tolerance involves different mechanisms of plant response and is present separately in each gene pool panel. Therefore, different phenotyping methods needed to identify relevant genotypes and traits. The genotypes presented here could be an excellent resource for parental selection and the two gene pools could provide complementary traits to improve the drought adaptation in common bean.

## Author contributions

AA and MB conceived the study, AA conducted experiments, AA, DA, and TS analyzed data. AA and MB wrote the manuscript, DA contributed to writing. All authors read and approved the manuscript.

### Conflict of interest statement

The authors declare that the research was conducted in the absence of any commercial or financial relationships that could be construed as a potential conflict of interest.
