# Supplementary material for: Trait Associations in Diversity Panels of the Two Common Bean (Phaseolus vulgaris L.) Gene Pools Grown under Well-watered and Water-Stress Conditions
Source: Front Plant Sci. 2017 May 9;8:733. doi: 10.3389/fpls.2017.00733 (PMC5422517; doi:10.3389/fpls.2017.00733)
Supplement: Supplementary file 1 [file Table1.DOCX]

**Supplementary Table 1**. Estimates of correlation coefficients between common bean traits (genetic below diagonal and phenotypic above diagonal with values in parenthesis indicating the standard errors) in Andean gene pool diversity panels grown under drought stress (DS) and non-stress (NS) treatments at Hawassa, Ethiopia, in 2009.

|  | Stress regime | YLDH | DF | DPM | SCMR | PDPL | SDPD | 100SW | PPI | PHI | HI |
| --- | --- | --- | --- | --- | --- | --- | --- | --- | --- | --- | --- |
| YLDH | DS | 1.00 | *-0.27*(0.09) | -0.06(0.03) | 0.03(0.04) | **0.39**(0.08) | -0.03(0.10) | **0.52**(0.04) | **0.42**(0.03) | *0.24*(0.02) | **0.44**(0.04) |
|  | NS | 1.00 | -0.03(0.10) | 0.11(0.09) | -0.06(0.07) | **0.30**(0.07) | 0.11(0.09) | **0.38**(0.03) | **0.33**(0.03) | 0.11(0.09) | **0.32**(0.04) |
| DF | DS | **-0.34**(0.11) | 1.00 | **0.52**(0.06) | *-0.25*(0.07) | **-0.34**(0.08) | *0.24*(0.08) | **-0.39**(0.08) | *-0.28*(0.03) | -0.10(0.08) | **-0.35**(0.08) |
|  | NS | -0.02(0.12) | 1.00 | **0.64**(0.05) | **-0.42**(0.07) | -0.19(0.07) | 0.17(0.09) | -0.14(0.10) | -0.11(0.10) | *-0.22*(0.08) | -0.10(0.09) |
| DPM | DS | -0.08(0.05) | **0.78**(0.07) | 1.00 | **-0.31**(0.07) | *-0.25*(0.08) | 0.20(0.08) | -0.20(0.09) | **0.29**(0.08) | 0.05(0.08) | *-0.22*(0.08) |
|  | NS | 0.13(0.13) | **0.88**(0.05) | 1.00 | **-0.38**(0.07) | -0.01(0.08) | 0.03(0.09) | 0.14(0.09) | -0.05(0.09) | -0.19(0.08) | -0.05(0.09) |
| SCMR | DS | 0.19(0.10) | **-0.57**(0.19) | **-0.52**(0.19) | 1.00 | 0.04(0.07) | -0.20(0.40) | 0.06(0.08) | 0.05(0.08) | 0.001(0.07) | 0.03(0.08) |
|  | NS | -0.09(0.11) | **-0.73**(0.09) | **-0.65** (0.11) | 1.00 | -0.03(0.07) | -0.08(0.08) | -0.08(0.09) | -0.03(0.09) | 0.16(0.08) | -0.04(0.08) |
| PDPL | DS | **0.52**(0.13) | **-0.49**(0.12) | **-0.46**(0.13) | 0.22(0.22) | 1.00 | **-0.29**(0.08) | **0.32**(0.08) | **0.55**(0.06) | -0.01(0.08) | **0.53**(0.06) |
|  | NS | **0.58**(0.17) | *-0.36* (0.18) | -0.22(0.20) | 0.06(0.22) | 1.00 | **-0.33**(0.07) | *0.26*(0.07) | 0.08(0.08) | 0.03(0.07) | 0.06(0.08) |
| SDPD | DS | -0.01(0.13) | **0.44**(0.12) | *0.30*(0.13) | **-0.59**(1.49) | *-0.35*(0.13) | 1.00 | -0.14(0.09) | -0.07(0.09) | *0.26*(0.07) | -0.03(0.09) |
|  | NS | 0.17(0.13) | *0.28* (0.12) | 0.13(0.14) | -0.13(0.15) | -0.30(0.18) | 1.00 | **-0.34**(0.08) | **0.46**(0.07) | 0.09(0.08) | **0.49**(0.07) |
| 100SW | DS | **0.53**(0.05) | **-0.52**(0.10) | **-0.32**(0.12) | 0.27(0.20) | **0.41**(0.12) | -0.21(0.13) | 1.00 | **0.45**(0.07) | *0.27*(0.08) | **0.47**(0.07) |
|  | NS | **0.42**(0.05) | -0.21(0.12) | 0.09(0.13) | -0.07(0.14) | **0.62**(0.17) | **-0.45** (0.10) | 1.00 | 0.07(0.10) | 0.14(0.09) | 0.08(0.09) |
| PPI | DS | **0.54**(0.05) | **-0.38**(0.05) | **-0.48**(0.11) | 0.18(0.20) | 0.70(0.08) | -0.04(0.14) | **0.60** (0.09) | 1.00 | 0.12(0.08) | **0.91**(0.01) |
|  | NS | **0.44**(0.04) | -0.15(0.12) | -0.04(0.13) | 0.01(0.15) | *0.45*(0.19) | **0.55**(0.10) | 0.10 (0.13) | 1.00 | 0.19(0.08) | **0.90**(0.02) |
| PHI | DS | **0.34**(0.04) | -0.11(0.17) | -0.01(0.18) | -0.01(0.27) | -0.11(0.19) | 0.50(0.15) | **0.52**(0.14) | 0.23(0.17) | 1.00 | **0.35**(0.07) |
|  | NS | 0.14(0.13) | *-0.37* (0.12) | **-0.42**(0.13) | 0.11(0.16) | 0.15(0.21) | 0.11(0.15) | 0.24 (0.13) | 0.20 (0.14) | 1.00 | **0.38**(0.07) |
| HI | DS | **0.56**(0.05) | **-0.47**(0.11) | **-0.44**(0.12) | 0.27(0.21) | **0.68**(0.09) | 0.02(0.14) | **0.64**(0.09) | **0.97**(0.01) | **0.41**(0.15) | 1.00 |
|  | NS | **0.46**(0.07) | -0.15 (0.13) | -0.04(0.14) | -0.03(0.15) | *0.41*(0.20) | **0.55**(0.10) | 0.14 (0.13) | **0.98** (0.01) | **0.42**(0.12) | 1.00 |

*Bold significant at 0.01 p value; italic and underlined significant at 0.05 p value

Abbreviations: DF, days to flowering (number); DPM, days to harvest maturity (number); SCMR, SPAD leaf chlorophyll meter reading (SPAD); PDPL, pods per plant (number); SDPD, seeds per pod (number); 100SW, hundred seed weight (g); PPI, pod partitioning index (%); PHI, pod harvest index (%); harvest index (%).
